# Generation of direct current electrical fields as regenerative therapy for spinal cord injury: A review

**DOI:** 10.1063/5.0152669

**Published:** 2023-09-19

**Authors:** Lukas Matter, Bruce Harland, Brad Raos, Darren Svirskis, Maria Asplund

**Affiliations:** 1 Department of Microtechnology and Nanoscience, Chalmers University of Technology, SE 41296, Gothenburg, Sweden; 2 Department of Microsystems Engineering, University of Freiburg, Georges-Köhler-Allee 201, GER 79110, Freiburg, Germany; 3 Brainlinks-Braintools Center, University of Freiburg, Georges-Köhler-Allee 201, GER 79110, Freiburg, Germany; 4 Freiburg Institute for Advanced Studies (FRIAS), University of Freiburg, Albertstraße 19, GER 79104, Freiburg, Germany; 5 School of Pharmacy, The University of Auckland, NZ 1023 Auckland, New Zealand; 6 Division of Nursing and Medical Technology, Luleå University of Technology, SE 97187 Luleå, Sweden

## Abstract

Electrical stimulation (ES) shows promise as a therapy to promote recovery and regeneration after spinal cord injury. ES therapy establishes beneficial electric fields (EFs) and has been investigated in numerous studies, which date back nearly a century. In this review, we discuss the various engineering approaches available to generate regenerative EFs through direct current electrical stimulation and very low frequency electrical stimulation. We highlight the electrode–tissue interface, which is important for the appropriate choice of electrode material and stimulator circuitry. We discuss how to best estimate and control the generated field, which is an important measure for comparability of studies. Finally, we assess the methods used in these studies to measure functional recovery after the injury and treatment. This work reviews studies in the field of ES therapy with the goal of supporting decisions regarding best stimulation strategy and recovery assessment for future work.

## I. INTRODUCTION

Spinal cord injury (SCI) typically causes long-term neurological impairments in humans. In addition to the often permanent loss of motor function and sensation below the levels of the injury, SCI typically involves other medical issues such as loss of bladder and bowel function, chronic wounds, frequent infections, and neuropathic pain.^1–6^ Typical causes of traumatic SCI are falls, motor vehicle accidents, and physical violence.^7,8^ About 50% of SCIs involve persons in their late teens and early twenties, with long life expectancies ahead of them.^9^ As the elderly also have a high prevalence of SCI, an increased incidence is expected with aging populations.^10^ Improved strategies to treat SCIs would have an enormous impact on improving the quality of life of individual patients and could in addition reduce the associated healthcare and socioeconomic costs.^11^

New possibilities with bioelectronic medicine have emerged that give hope for better treatments for patients living with a SCI. Functional electrical stimulation could be a tool to modulate motor activity patterns and restore motor functions.^12–18^ Electrical stimulation (ES) furthermore is used to reduce chronic pain.^19^ A long-term and highly challenging goal is to achieve regeneration of injured nerve pathways in the spine, to restore lost functions as well as sensation. Next to explorative regenerative therapies including neural cell transplantation^20–22^ and pharmacological treatments,^23,24^ ES could have a role in supporting the regenerative processes itself. Electrical fields (EFs) have been proposed as a potential guidance signal that could increase the chances of constructive axonal regeneration and reinnervation. Direct current electrical stimulation (DC-ES) has been found particularly effective to promote and guide axonal growth, thereby reconnecting injured fibers and restoring function.^25,26^ DC-ES generates an EF across the tissue, which in cell culture and in animals has been shown to cause axons to grow toward a cathode (negative pole), while regressing from an anode (positive pole).^27–30^ Importantly, growth toward the cathode was found to be faster than die-back from the anode in the initial period (∼min) of a pulse, which led to a very low frequency electrical stimulation (VLF-ES, < 1 Hz) being proposed as a viable therapy.^31^ In VLF-ES, the EF polarity is reversed, typically every 15 min, to promote axon outgrowth in opposite directions, thus, more effectively regenerating sensory and motor fibers.

To date, the majority of reviews focusing on DC-ES and VLF-ES are about outcomes of *in vitro* and preclinical studies and the underlying cellular mechanism of ES-promoted axon growth.^26,32,33^ However, the design of the system to deliver DC is critical. It is important to note that direct current (DC) is involved in both DC-ES and VLF-ES, which is challenging to the electrode material. To supply DC, charge is injected by redox reactions, which might be irreversible and generate cytotoxic by-products as well as induce local pH changes.^34–38^ Better solutions are in development, e.g., replacing metals with unconventional electrical materials such as conducting polymers (CPs) with large ionic pseudo-capacitance and improved capability to supply DC in a biocompatible fashion.^39,40^ This means that they can supply DC while in close contact with tissue, without inducing toxic side-effects.^41,42^ This in turn improves the possibilities to supply precision ES from implants in intimate contact with the spinal cord. Furthermore, fabrication and design technology has advanced. Computer aided design in combination with modern imaging techniques improve optimization of desired device functionality by allowing implants to be tailored to the anatomical location.^12–14,18^ Employment of soft or thin materials ensures an implant that conforms to the curvature, thus reducing the risk of compression.^43–46^ Taken together, these advancements increase the biocompatibility of bioelectronics implants so that electrodes can be placed closer to the tissue to be treated, which in turn increases the spatial precision of the generated EF and hopefully also the effectiveness of the therapy.

These recent developments are promising and call for intensified efforts in translating results to clinical application.^33,47^ One challenge for the field is that the published data, although similar in the aims set out (using DC stimulation to promote regeneration), represents a diversity of experiments. From this diverse dataset, it is not always easy to deduce which parameters were used and which were found effective.^26,32^ Biological tissue is heterogenous, which contributes to natural large variability in the EF strength and more specifically in the expected current distribution in the tissue.^48–50^ In addition, the electrodes, their placement, as well as the control circuitry powering the stimulation will influence the EF.^51–53^ Furthermore, electrochemistry on the surface of the electrodes will influence the biochemical microenvironment adjacent to the electrodes, which in turn might have a negative impact on the outcome.^34–37^ Although the concept of electrical stimulation is straightforward, the implementation and interpretation is a complex and multi-faceted challenge.

This review aims to provide an overview of the chosen engineering approaches to deliver DC *in vivo* including methods to estimate field strength. Techniques used to evaluate recovery following damage and therapy are described and evaluated in order to connect the engineering approach with functional recovery. With the aim to support more translational efforts in this promising field, each section of this work begins with a brief introduction for scientists from other disciplines. At the start, we describe the electrode–tissue interface, methods for characterization, and show that the electrode surface is altered by electrical stimulation in saline. The degree of alteration depends on the electrode material and the electrochemical stress, which we explore in Secs. III and IV, respectively. In order to sustain the stimulation, the electronics need to be designed accordingly. We discuss the various system approaches used in the literature in Sec. V. Section VI introduces methods for EF estimation including a finite element model of a rat's spine during DC-ES and a summary on electrode placement and reported EF strength in the literature. Approaches to assess treatments and identify the potential beneficial effect of EFs on regeneration are discussed in Sec. VII. Finally, clinical translation of these technologies is briefly discussed in Sec. VIII.

## II. ELECTRODE–TISSUE INTERFACE

In order to generate an electric field in the target tissue, electrodes need to be placed close to the area to be stimulated. As a simplification, the magnitude of the field at a specific point in the tissue decreases with the inverse square of the distance to an electrode. The electrodes will, electrochemically speaking, constitute a phase boundary, where electronic charge carriers in the solid state are converted to ionic charge carriers in the tissue.^54^ For this to occur, electrochemical mechanisms are needed on the electrode surface. These reactions (e.g*.,* oxidation of metal electrode) will supply ions, but may generate cytotoxic byproducts or may change the surrounding environment (e.g*.,* pH).^34–38^ To minimize such side-reactions, it is important to carefully consider the interplay between stimulation applied and the electrode material, in other words, the electrochemistry at the electrode–tissue interface. Electrochemical characterization methods can here be helpful to assess electrode degradation during and after the therapy. In this section[Sec s3], the theory of the analysis is explained. Then, common electrode materials are reviewed. In addition, we provide an *in vitro* stimulation example to show the benefits of assessing the electrode status by electrochemical analysis prior to *in vivo* work.

**FIG. 1.**
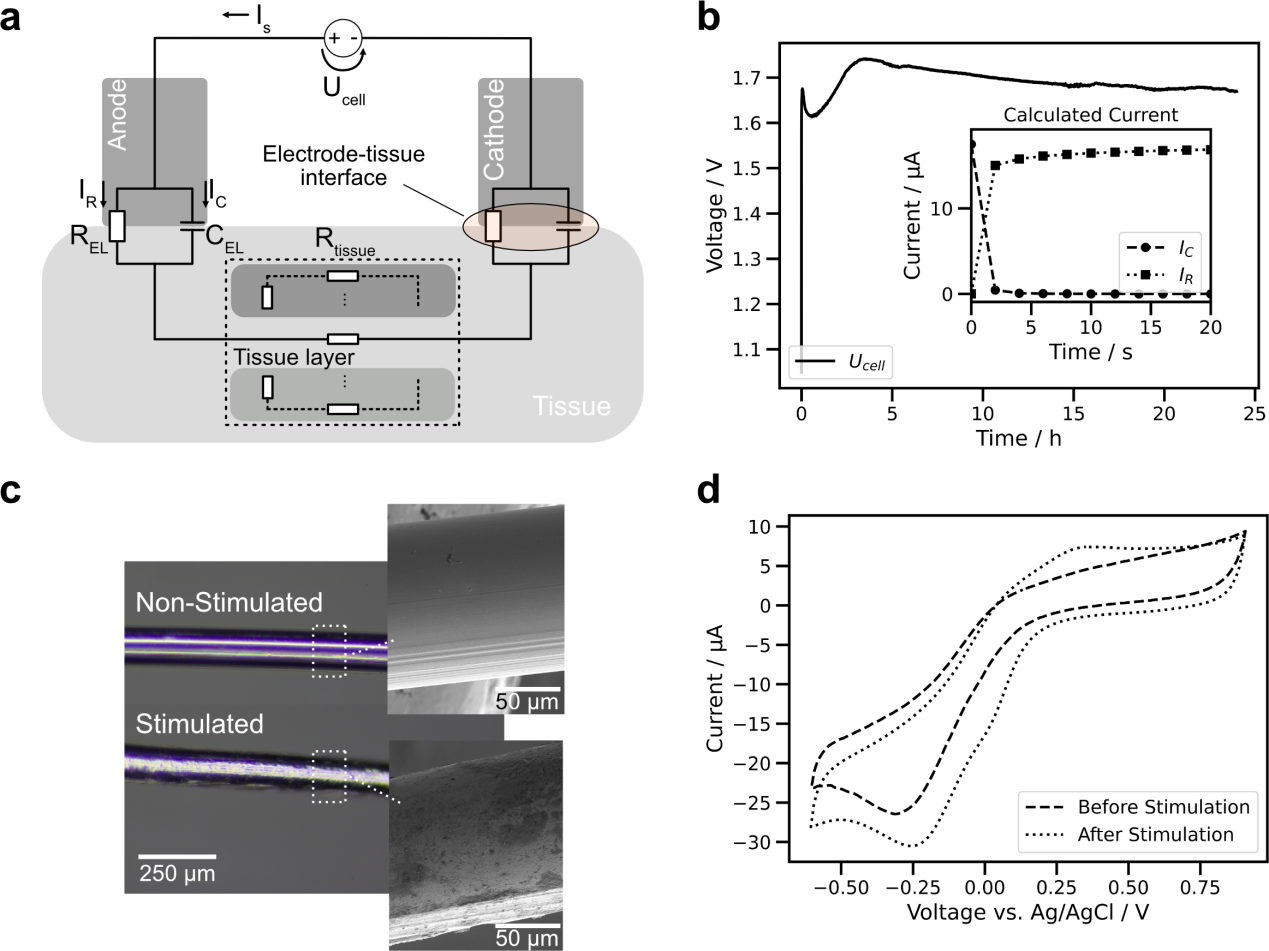
(a) The equivalent circuit of a common stimulation setup consists of electrodes [ $R_{EL}(t)$ and $C_{EL}(t)]$ in contact with tissue [ $R_{\text{tissue}}(t)$]. The stimulating current $I_{S}$ is generated by an energy source $U_{\text{cell}}(t)$. The resulting electric field strength depends on the characteristics of the respective tissue layers. (b) Recorded voltage $U_{\text{cell}}(t)$ of the stimulation experiment. $U_{\text{cell}}(t)$ increases rapidly, which is typical for capacitive current $I_{C}(t)$. Afterward the voltage stabilizes, speaking for a predominantly faradaic current $I_{R}(t)$. The inset shows the estimated time dynamics of capacitive and faradaic current based on the slope of $U_{\text{cell}}(t)$. (c) Images of 5 cm PtIr wire (0.18 mm diameter) as anode before and after DC stimulation (24 h, 17.5 *μ*A). (d) Comparison between the cyclic voltammograms (CV, 100 mV/s) collected with the wire before and after stimulation reflects the surface change. After stimulation, an oxidation peak at 0.3 V and an increased reduction current at −0.25 V are present. We interpret the former as oxidation peak from Ir, while the latter might speak for a higher density of oxides from PtIr.^62^ The changes express that after the stimulation experiment the electrode undergoes reduction and oxidation more effectively as seen in the increased/decreased current for positive/negative peaks. Further experiments are necessary for a precise analysis of the changed composition of the PtIr wire. The applied current was similar to the work of Borgens *et al.*^53^

In the simplest case, a stimulation setup consists of two electrodes separated by tissue, where tissue will fill the role of an electrolyte [Fig. 1(a)]. The electrodes inject charge carriers into the electrolyte by the means of one or more electrochemical mechanisms. These mechanisms can be categorized as Faradaic reactions and non-Faradaic processes. Faradaic charge transfer is the result of electrochemical reactions, for example, the oxidation of a metal electrode. The specific charge transfer mechanisms that are available depend on the materials and properties of the electrodes and the chemical composition of the electrolyte. Non-Faradaic processes are primarily associated with the ionic double layer (Helmholtz double-layer), which forms due to the accumulation of ions at the electrode surface comprising the so-called Helmholtz capacitance. Thus, charge is transferred by the reorganization of the charged species in the tissue, which generates an electrical current. For porous electrode materials, there can furthermore be an additional bulk-contribution to electrode capacitance by ions stored inside the porous material. In a simplified equivalent circuit model, it is common to model the electrodes (anode A and cathode C, respectively) as a resistor $R_{EL}(t)$ accounting for faradaic reactions in parallel to a capacitor $C_{EL}(t)$ [Fig. 1(a)]. This circuit is here referred to as an −RǁC− element. The tissue is represented by the resistance $R_{\text{tissue}}(t)$. $R_{EL}(t)$, $C_{EL}(t)$, and $R_{\text{tissue}}(t)$ change over time t because of various reasons including tissue reactions such as scaring or changes at the electrode–tissue interface, e.g*.,* through faradaic reactions.^55–57^ In the simplified equivalent circuit in Fig. 1(a), the tissue is considered as a single resistance. In reality, the tissue is more complex, and different layers of the tissue need to be taken into account [as implied by parallel resistances in Fig. 1(a)]. Also, the charge transfer at the electrodes is more complex than an −RǁC− element, and a multitude of models can be used to more precisely mimic the electrode–electrolyte interfaces and their dependency of physical phenomena, such as diffusion.^57–59^

The dynamic and magnitude of the voltage drop over an electrode gives information about the nature of the electrochemical mechanisms taking place. When a constant current $I_{S}$ is driven through the system the voltage over the different components can be calculated by Ohm's Law U=RI, which leads to $U_{EL}^{A}(t)$, $U_{EL}^{C}(t)$, and $U_{\text{tissue}}(t)$ over anode, cathode and tissue, respectively. The sum of these voltages is here defined as $U_{\text{cell}}(t)$. The electrical current is transported through the electrolyte by ionic charge carriers, which will be driven by the charge unbalance created at the electrode surfaces. Positive species are released at the anode, negative at the cathode, and ions travel toward the cathode/anode, respectively. Typically, the current $I_{C}(t)$ through $C_{EL}(t)$ decreases over time, and, consequently, the current $I_{R}(t)$ passing through the resistance $R_{EL}(t)$ increases [Fig. 1(b)]. From an electrochemical viewpoint, charge transfer after a certain amount of time will mostly happen through faradaic reactions, because the Helmholtz double layer capacitance will be fully charged and not pass any further current. This means that the capacitance at the surface of the electrode is fully occupied by ions. Hereby, the initial capacitive charging leads to a fast change of $U_{\text{cell}}(t)$ compared to the faradaic reactions as shown in the big initial slope in $U_{\text{cell}}(t)$ in Fig. 1(b). One example for a faradaic reaction, which is determined by the magnitude of $U_{EL}^{A}(t)$ and $U_{EL}^{C}(t)$, is the electrolysis of water, which results in the formation of oxygen or hydrogen gas and changes pH of the surrounding electrolyte. The threshold potentials where this happens depend on the material and the electrolyte composition, but for Pt-based implants, it is commonly set as 0.6 and 0.9 V (voltage vs Ag/AgCl reference), also referred to as the “water window.”^54^ Since water is available in abundance in tissue electrolysis occur at high rate when $U_{EL}^{A}(t)$ or $U_{EL}^{C}(t)$ exceeds the water window. Thus, these boundaries set the upper and lower thresholds where major stimulation-driven damage to electrodes and tissue can be expected. In practice, more conservative limits are used as reactions other than electrolysis also may occur even when the potential is kept well within the water window.^37,60^

Although endogenous electrical fields occur it can be assumed that, when no current is applied, the distribution of ions is uniform ( $U_{\text{tissue}}=0$). $U_{\text{cell}}$ then equals the sum of Nernst potentials also referred to as “electrode potentials” at anode and cathode. The Nernst potential at the anode and cathode in the no current case is the potential difference between the electrode material and the surface, which is in contact with the electrolyte. The applied voltage needs to be larger than $U_{\text{cell}}$ ( $I_{S}=0$) in order to drive electrochemical reactions.^61^

Values of the elements of the −RǁC− circuit introduced in this section depend on various factors such as the electrode material. Some materials only support capacitive charge exchange (e.g., titanium nitride), while others mainly rely on faradaic reactions (e.g., Ag/AgCl). According to the dominant charge exchange mechanism, the representative elements in the model or the applied model itself change. Additionally, specific electrode materials may catalyze certain electrochemical reactions (e.g., oxygen reduction).^60^ These factors need to be considered to apply safe electrical stimulation. Electrode materials utilized in the application of direct current to the spinal cord are explored in Sec. III.

## III. ELECTRODE MATERIALS

The potential at which certain electrochemical reactions occur is material and electrolyte dependent. For *in vivo* DC application in the spinal cord, three electrode materials have been used to date in the literature: silver–silver chloride (Ag/AgCl),^29,63–65^ platinum–iridium (PtIr),^53,66–83^ and stainless steel.^30,84^ Ag/AgCl electrodes are non-polarizable ( $C_{EL}\approx 0$), meaning that current is mainly generated through faradaic reactions. The redox reaction on their surfaces follow:

$$\text{Ag}\left(s\right)⇌{\text{Ag}}^{+}+{\;e}^{-},$$ (1)

$$\text{Ag}\left(s\right)\;+{\;\text{Cl}}^{-}⇌\text{AgCl}\left(s\right)\;+{\;e}^{-}.$$ (2)

This means, passage of current results in a release of silver ions at the anode, making the stimulation cytotoxic.^85,86^ In order to inhibit the transport of silver ions to the tissue, electrodes can be constructed as wick electrodes, in which the Ag/AgCl electrode is at one side of an electrolyte filled tube containing a centimeter-long cotton thread, with the tissue being on the other side.^29,63–65^ Eluted silver ions migrate through the cotton thread over time both due to passive diffusion, but also driven by the electric field. Once the buffer capacity of the system (electrolyte plus cotton thread) is exhausted, the ions will leak into the tissue. Thus, the longer the stimulation or the higher the current, the higher the concentration of accumulated silver ions in the tissue.^38^ It should be noted here that if no cotton thread was applied, the silver ions would of course elute immediately into tissue.

In subsequent work, Ag/AgCl wick electrodes were substituted by PtIr electrodes to avoid the strong toxicity of silver ions.^66^ Either a PtIr wire^53,66–81^ (PtIr, typically 90/10%) was implanted, which was coiled in some studies, to increase the electrodes' surface area^53,66–78,81^ or PtIr disk electrodes.^82,83^ Here, PtIr was in direct contact with tissue. In contrast to Ag/AgCl, PtIr has the capability to inject charge both by non-faradaic processes and faradaic reactions.^35–37,87^ For DC stimulation, it is nevertheless only faradaic reactions that will come into question, as the capacitive charge transfer will be exhausted within minutes. If the potential exceeds the water-window, the dominant reaction will be electrolysis, but there are also possible reactions for Pt electrodes within the water window, in particular, metal oxidation, such as

$$\text{Pt}\;+{\;4\;\text{Cl}}^{-}⇌{\text{PtCl}}_{4}{}^{2-}+{\;2e}^{-}.$$ (3)

PtCl_4_^2−^ acts as oxidizing agent and was shown to be cytotoxic.^88,89^ The human body contains the chloride needed for this reaction, e.g., in cerebrospinal fluid.^90^ Dissolved platinum is toxic, but is generated in lower rate than platinum chlorides.^91–93^ The amount of Ir in PtIr is small (10%), which is why we assume that its influence on the release of cytotoxic products during stimulation is not significant. That being said, the toxicity of Ir ions and compounds released during stimulation has not yet been thoroughly investigated. However, oxidized Ir (IrOx) has been successfully used as electrode material in animals without signs of toxicity after pulsed stimulation (*μ*s–ms pulse widths).^94–97^ In general, Ir has a higher charge injection capacity than Pt due to different oxidation states, which remain bound to the electrode surface.^87,98,99^ The cytotoxic effect of platinum compounds generated by ES explains why in the work of Hurlbert *et al.*,^100^ it was found that stimulation with PtIr electrodes caused demyelination and harmful pathological changes. This is also in agreement with observations of tissue damage after DC stimulation in dogs.^69^ It should be noted that also in a well-established application such as cochlear implants, Pt corrosion over time is a factor that increases the foreign body reaction.^101^

Alternatively, to PtIr wires, sometimes stainless-steel wires were implanted.^30,84^ Oxidation of stainless steel leads to the release of iron, chromium, and manganese ions.^102^ Compared to bare platinum electrodes, stimulation with stainless-steel electrodes is reported to be more cytotoxic.^103^ In most studies, one or both electrodes were positioned within the muscle, which is to limit damage to the spinal cord itself.^53,69,71,73,74,78,81,84,104^

In the field of bioelectronics, common other electrode materials with promising capacity for DC-ES are IrOx, carbon nanotubes, and CPs.^34^ Various processing techniques are available to fabricate IrOx electrodes (i.e., sputter, thermal, and electrochemical). Depending on the fabrication method, the electrodes have different characteristics. However, the reason why IrOx, in general, is promising for DC-ES is its capacity for pseudocapacitive charge injection. Pseudocapacitive processes [i.e*.,* Ir(III)/Ir(IV) transition] are primarily based on intercalation or adsorption of ions at the surface of the electrode, thus, keeping reaction by-products bound to the electrode surface.^62,105^ Surface bound ions, therefore, do not diffuse into the target, which minimizes the influence of the ES on the chemical composition of the tissue. Carbon nanotubes provide a large surface area. The time of capacitive current scales with surface area, which makes carbon nanotubes and nanofibers attractive for DC-ES. Through functionalization of carbon nanotubes, their properties can be enhanced (i.e., biocompatibility), which may be especially interesting for electrodes in contact with the delicate spinal cord.^106–108^ CPs are promising for DC-ES because they have the potential to contain mobile ions (e.g., intrinsically or absorbed from electrolyte) within their polymer matrix. When available, the mobile ions may be injected or ejected during stimulation depending on the electrode polarity. The mobile ions within the polymer matrix contribute to an increased capacitance prolonging the time of non-faradaic charge injection.^109^ In the category of CPs for DC-ES, poly(3,4-ethylenedioxythiophene) (PEDOT) and its various anionic dopants (e.g., polystyrene sulfonate, dodecylbenzenesulfonate) have gained most attention because of their high electrochemical stability, flexibility, and ease of processing.^57,110,111^ The most common dopant for PEDOT is polystyrene sulfonate (PSS), because it results in good mixed ionic-electronic conductivity and processability.^112^ In combination with IrOx as base electrode, electropolymerized PEDOT/PSS shows high stability and withstands DC-ES.^39,113,114^ However, the charge injection mechanism of IrOx, carbon and PEDOT are not fully understood. Especially characterizing the generated species, their diffusion length, and cytotoxic effect during DC-ES would be beneficial for the field. A future application of CPs in spinal cord regeneration is their capability to be loaded with bioactive compounds, both proteins and smaller drug molecules.^115,116^ These bioactive compounds, which may be passively released over time or actively during stimulation, can be tailored for various purpose such as minimizing the foreign body response or altering the biological environment of the central nervous system to enhance its naturally poor regeneration capacity.^117,118^ Furthermore, we see that implantable systems for electrophoretic drug delivery to the spinal cord would be a possible pathway to enhance ion delivery capabilities so that electrical field could be maintained for longer. Such systems have been developed for delivery of the neurotransmitter g-aminobutyric acid but has, to the best of our knowledge, not been explored for the purpose of achieving DC fields.^119^

In summary, application of DC is dependent on electrochemical reactions at the electrode surfaces to inject the charge, and this mechanism will result in a change to the electrolyte (i.e*.,* the tissue). Thus, it is very important to consider in which way the specific electrode material generates the electrical current in the tissue, and specifically which ions will be involved, as these may be toxic or have substantial impact on the local pH. Clearly, a biocompatible DC stimulation electrode material would improve the possibility to stimulate the spinal cord with electrodes placed in immediate contact, likely decreasing the current needed and increasing precision of the method. In order to compare the effect on the electrode from different stimuli, the concept of electrochemical stress is discussed in Sec. IV.

## IV. ELECTROCHEMICAL STRESS

One way to compare the extent of electrochemical stress imposed on an electrode of a certain size, by different pulse-duration and amplitudes, is to compare the resulting charge density $\sigma_{P}$ per pulse. For DC applications, $\sigma_{P}$ is high because currents are applied continuously over hours up to weeks. A common stimulation pattern for SCI regeneration is VLF-ES with pulse widths of 15 min. Initially, at the leading edge of the pulse, the capacitance will charge/discharge. Nevertheless, the capacitive mechanism is quickly exhausted. The reported charge injection limit per pulse for PtIr (20% Ir) wires, which is taking advantage of capacitive current during short (200–400 *μ*s) pulses, is 0.3 mC cm^−2^.^98^ As a comparison, the limit for IrOx was reported as approximately 4 mC cm^−2^.^99^ Note that the charge injection limit is reached when the cell voltage is above the water window. Short pulses draw proportionally larger advantage of the capacitive charge injection, whereas the capability under DC conditions is much lower. In Table I, the applied charge and the size of utilized electrodes of the reviewed studies (where information was available) are summarized. For a typical DC-ES study, $\sigma_{P}$ is in the range of 220 mC cm^−2^–22.7 kC cm^−2^. For VLF-ES, it is a maximum of 3.8 C cm^−2^. For all studies, the capacitive limit of charge injection per pulse for PtIr is exceeded by more than a factor of 1000, even in the most conservative case of VLF-ES. Assuming that for water electrolysis, damage to the electrode surface and generation of cytotoxic by-products are tolerable side-effects, it is in principle possible to stimulate above this threshold. We tested DC stimulation with a similar approach as in Borgens *et al.*^53^ We found that the applied stimulus induced visible changes of the electrode surface already after 24 h of stimulation, also indicated by a change in the cyclic voltammograms (CV) [see Figs. 1(c) and 1(d)]. In this case, it can be assumed that the change in the surface is a mixture of electrochemical corrosion of platinum, possibly accelerated by an electrochemically driven reduction of pH, as well as mechanical damage caused by bubbles forming because of electrolysis.

**TABLE I.** Injected charge and electrode area (C = cathode, A = anode) of the reviewed studies if reported (/ for not reported). If a range of experimental days was provided, the lower limit was used for charge calculation. For VLF-ES, delivered charge is smaller because the direction of the applied current is reversed every 15 min.

Material	Charge (C)	Electrode area (mm^2^)	Charge Density per pulse $\sigma_{P}$ (C cm^-2^)	References
DC-ES				
Ag/AgCl	4	/	/	63, 65
	44	/	/	29
	121	/	/	64
PtIr	68	2 (C), 0.3 (A)	3.4 k (C), 22.7 k (A)	66
	5.4	0.6	0.9 k	67, 68
	121	57	0.21 k	53
	121	28 (C), 57 (A)	0.42 k (C), 0.21 k (A)	70
	140	/	/	84
	0.3	0.04 (C), 136 (A)	0.75 k (C), 0.22 (A)	30
VLF-ES				
PtIr	0.18	/	/	69, 71
	0.18	4.72	3.8	73, 74
	0.01	/	/	75, 76
	0.04	19	0.2	78, 81
	0.04	/	/	72
	0.05	/	/	79, 80

The electrochemical stress in this section was calculated under the assumption of a constant current over the period of the experiment. An electrical stimulator is the entity providing this constant current. The stability of the applied current depends on the stimulator circuitry. The stimulator is, therefore, a crucial part of every DC-ES experiment. The various stimulator circuits utilized in the reviewed studies are discussed in Sec. V.

## V. STIMULATORS

The current driven though the electrodes is controlled by a stimulator, which is the electronic circuitry that controls a power source to drive a specific stimulus. It is important that the employed stimulator circuit is able to generate the required current over the time of the therapy, meaning that the implementation must be such that it adapts to changes in impedance of the electrode and tissue. While a change in electrode impedance may occur due to altering of the electrode surfaces, e.g., because DC stimulation driven redox reactions,^37^ a change in the tissue′s impedance is a consequence of the biological response after the implantation process.^55,56,120^ The reviewed studies used a variety of different approaches to account for the variability in impedances. One way to mitigate variability in tissue and electrode impedance is to add a high Ohmic resistor, several orders of magnitude higher than the expected tissue resistance, such that the variability is comparably small. Similarly, a transistor with high input impedance is utilized to decouple the current source from tissue and electrode impedance. In this section, the electrical theory of designing a stimulator is explained in brief. Then, utilized circuits and their parameters are explored. The circuitry to deliver DC and VLF-ES are similar, besides a timing element required for VLF-ES, so they are considered together in the theory paragraph.

Typical stimulators consist of an energy source connected to electrodes. For VLF-ES, additional timing elements are installed to switch the direction of the current, usually after 15 min in previous literature. An ideal energy source can either generate a constant voltage or current. In reality, the output of the energy source depends on the connected load, and a constant output can only be provided for a certain Ohmic load range. A battery is a common constant voltage source described by the output voltage $U_{0}$. A DC source delivering $I_{S}$ usually consists of a voltage source connected to a resistor $R_{in}$ or a transistor T. For the former, the current equals $I_{S}=U_{0}/Z_{\text{circuit}}(t)$. $Z_{\text{circuit}}(t)$ is the complex impedance of the circuit accounting for time-dependent interrelations between input and output voltage in magnitude and phase. One part of $Z_{\text{circuit}}(t)$ is the combined resistance of electrodes and tissue $Z_{\text{cell}}(t)$. When $Z_{\text{circuit}}(t)$ changes, the generated current varies. Usually, $R_{\mathrm{in}}$ is multiple magnitudes larger than $Z_{\text{cell}}(t)$, which increases the required $U_{0}$, but, on the other side, compensates for changes in $Z_{\text{cell}}(t)$. An increased $U_{0}$ poses the challenge of battery size, which usually scales with $U_{0}$, e.g*.,* by connecting multiple batteries in series. In order to keep the size of the required battery small, $Z_{\text{circuit}}(t)$ needs to be minimized. This is done by using a transistor to decouple $Z_{\text{cell}}(t)$ from the generated current. The transistor amplifies the current on its input, which is set by a resistor $R_{\text{set}}$, and therefore is independent from $Z_{\text{cell}}(t)$ over a wide range of resistances. $U_{0}$ needs to be chosen carefully so that a $U_{\text{cell}}(t)$ is required to generate $I_{S}$, thus practically limiting the stimulating current that can be applied. Therefore, a DC source will deliver the set current under the condition that $U_{0}$ is sufficient. In practice, the so-called voltage compliance gives information about the maximum voltage a current source will reach to deliver the set current. The voltage compliance is $U_{0}$ minus the voltage drop over the energy supplies internal electrical components. By knowing the stimulation current, the generated field strength can be estimated. This presents one of the reasons why in most studies a constant current source is installed in the stimulator instead of a constant voltage source. The application of a constant voltage will not result in a constant current or field strength, because $Z_{\text{circuit}}(t)$ varies over time. This is similar for $U_{\text{cell}}(t)$ if DC is applied as shown in Fig. 1(b). We discuss field estimation in Sec. VI in this review.

In Table II equivalent circuits for the different stimulators from the reviewed studies are shown. Roederer *et al.*^65^ manually controlled a voltage source to generate the required current, while in other works, a high Ohmic resistor $R_{\mathrm{in}}$ was connected in series to a voltage source [see Table II, circuits (a) and (b)].^63,67,68^ Knowing $R_{\mathrm{in}}$ and $U_{0}$ in circuit (b) of Table II allows us to calculate the maximum generated current by applying Ohm's Law, which results in 15 *μ*A and 3.2 nA for the two sets of parameters provided. The most common stimulator setup was a voltage source connected to a transistor over $R_{\text{set}}$ [see Table II, circuits (d) and (e)].^29,30,53,66,70,82–84^ For VLF-ES, additional timing elements depicted as switches in the circuits (d) and (e) of Table II were necessary. Similar to DC, either a voltage source connected in series to $R_{\mathrm{in}}$^72,75,76,78,81^ or a transistor^69,71,73,74^ was considered as stimulator. Interestingly, all reviewed works after 2014 have chosen to employ the former circuit to apply VLF-ES.^75,76,78,81^

**TABLE II.** Stimulator circuits of reviewed studies if stated.

	Circuit	Parameters	Functionality test	References
(a)	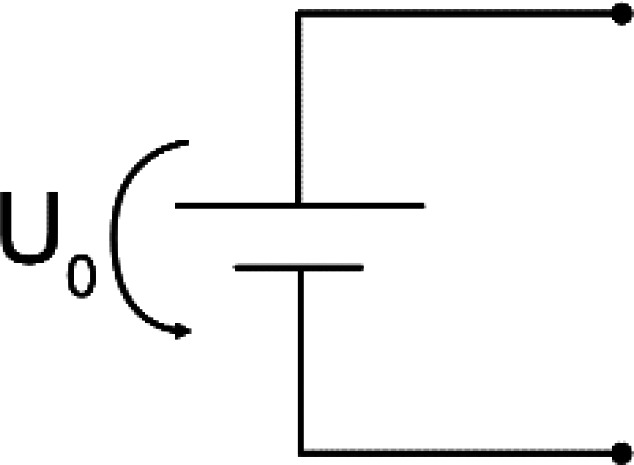	$U_{0}=60–70\;V$	Amperometer *in vivo*	65
(b)	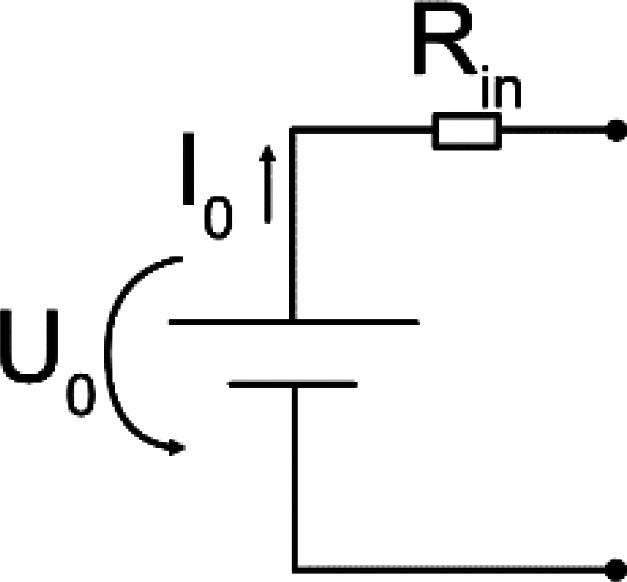	$U_{0}=30\;V,\;R_{\mathrm{in}}=2–4\;M\Omega$	Amperometer *in vivo*	63
		$U_{0}=1.5\;V,\;R_{in}=470\;M\Omega$	Amperometer after termination	67, 68
(c)	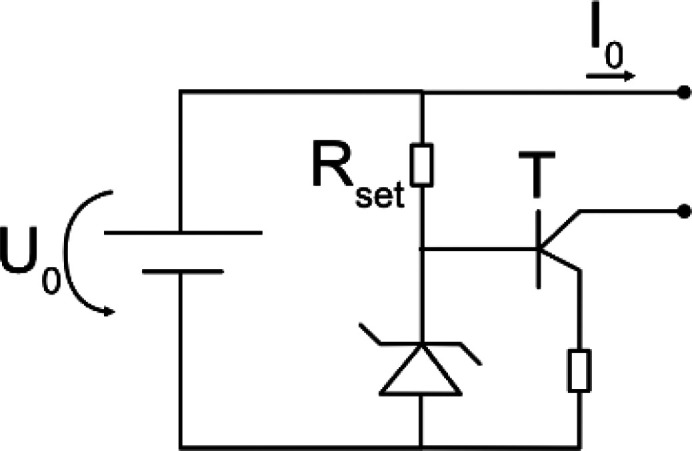	$U_{0}=9\;V,\;I_{0}=10\;\mu A$	After termination	29
		$U_{0}=6\;V,\;I_{0}=14\;\mu A$	Amperometer after termination	66, 82, 83
		$U_{0}=6\;V,\;I_{0}=30–50\;\mu A$	Voltmeter over known resistor^53^	53, 70, 84
		$U_{0}=3\;V,\;I_{0}=0.1\;\mu A$		30
(d)	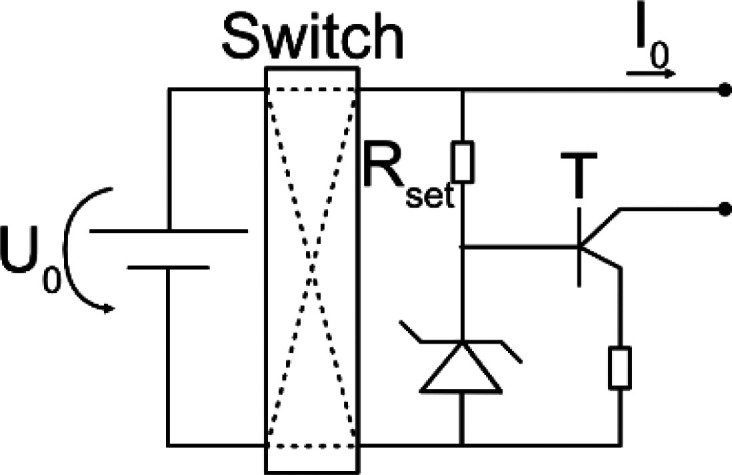	$U_{0}=6\;V,\;I_{0}=200\;\mu A$	Visual inspection after termination	69
		$U_{0}=3.6\;V,\;I_{0}=600\;\mu A$	Electrically after experiment^73,74^	71, 73, 74
(e)	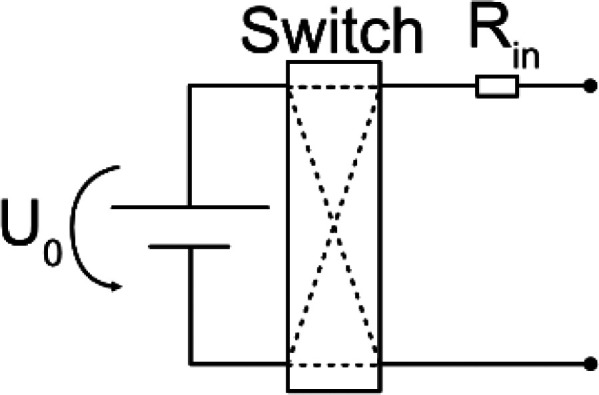	$U_{0}=3\;V,\;R_{\mathrm{in}}=30\;k\Omega$		72, 78, 81
		$U_{0}=3.3\;V,R_{\mathrm{in}}=130\;k\Omega$		75, 76

The circuit layout to generate DC is one aspect of the stimulator design. The longevity of the device delivering the required current over months is equally relevant. As previously mentioned, the electrode–tissue interface changes over time, which means the electrical load of the simulator circuit will vary. Testing the resilience of the system to impedance variations *in vitro* prior to the experiment or monitoring the generated current *in vivo* are two methods to ensure keeping the stimulation parameters as initially planned and safe. Before implantation, the stimulator can be assessed by delivering the stimulation, e.g*.,* into saline solution and by varying the resistive load to the output of the stimulator. While the former ensures that the electrode surfaces withstand the stimulus, the latter simulates the variability in $Z_{\text{cell}}(t)$. This is especially relevant for stimulation, which leverages redox reactions that may be irreversible, leading to slow dissolution of the electrode material. Both types of experiments are mentioned in the work of Fehlings and Tator *et al.*,^82^ Fehlings *et al.*,^83^ and Bacova *et al.*^79^ In Table II, we have summarized the techniques typically used in the field to evaluate the functionality of the stimulator during an experiment. For non-implanted energy sources, it is straightforward to monitor the output current using an amperometer.^63,65^ For fully implanted stimulators, other methods need to be considered such as measuring the voltage drop over two wires soldered onto each stimulation electrode,^83^ evaluating the voltage drop over a known resistor^53^ or implanting additional current probing electrodes.^79^ If the functionality could not be monitored during the experiment, then stimulators were visually^69^ examined, i.e*.,* by identifying corrosion of PtIr stimulation wires, or electrically^66–68,73,74,82,83^ tested after termination. In Wallace *et al.*,^66^ only 1 of the 9 stimulators were found functional (i.e*.,* capable to deliver a current) after termination of the experiment. If functionality of the system is not evaluated during or after an experiment, failure would go unnoticed, and it could be the case that in fact no stimulation was delivered, or stimulation was only applied at the very beginning. Further concerns with a non-monitored stimulation system is that electronics failure could result in an overload heating of the devices, or an excessive strong current leading to tissue damage. For these reasons, in addition to functionality tests, a fail-safe circuit for the stimulator was established in preparation for human trials.^71^ The stimulator implanted in dogs would switch the stimulation off in the case of an abnormal output, which was monitored by measuring the output voltage. In case the output voltage drops under a certain threshold, the connection to the energy source would be interrupted. This stimulator design was then further used in human trials.^73,74^

The aim of an appropriate choice of electrode material and stimulator is to generate an EF in the spinal cord. It is the EF's field strength that shown to have a therapeutic effect. Hence, it is important to inform about the applied EF dose. In Sec. VI, different methods to estimate the generated EF strength are discussed.

## VI. FIELD ESTIMATION

DC-ES has been experimentally tested in a variety of animal models showing promising treatment results (see Table III). However, to compare different studies, it is important to focus on what field strength was effectively generated in the target tissue acting on regrowing axons. In the literature, three methods to estimate the generated field can be found. First, equations derived from circuit theory are applied to estimate the field strength. Second, the same equations are utilized but values are directly measured in the stimulated tissue, e.g., resistance or voltage is monitored with a dedicated set of electrodes in contact with the tissue, which typically is done before closing the wound. The third approach is to estimate the field in the tissue by finite element modeling (FEM). At first, we present the theory of the first two approaches and compare it to results of FEM to explore limitations of field estimations. Furthermore, we provide an overview of the reported efficient field strengths in the literature.

**TABLE III.** Field strength, duration of stimulation and biological effects of reviewed literature if reported (G: guinea pig; D: dog; R: rat; H: human; HS: hemisection; CT: contusion; CP: compression; P: piercing; and N: naturally occurring SCI). ^i^ A detailed discussion of the reported field strength is found in Sec. VI. ^ii^ Based on battery capacity of 2400 mAh and stimulation current of 600 *μ*A.

Field strength (mV mm^−1)i^	Animal (injury) model	Duration	Biological effects	References
DC-ES				
0.04	G (HS)	50–60 days	-Histological: horseradish peroxidase dyed axons grew into and around glial scar at the plane of transection.	29
0.27 (35 *μ*A)	G (HS)	40 days (35 *μ*A)	-Behavioral: recovered skin reflex of the cutaneous trunci muscle (CTM) reflex in 9 of 67 animals in the cathode rostral group.	53
0.35 (50 *μ*A)		30 days (50 *μ*A)		
0.35	G (HS)	40 days	-Behavioral: delayed application of ES after injury (100 days) did not recover CTM reflex.	70
0.40	G (HS)	30 days	-Histological: fine axon processes crossed the plane of transection.	84
70	R (CP)	56 days	-Behavioral: increased score in inclined-plane task (53-gm weight drop injury group).	83
			-Histological: increased number of cells in red nucleus, raphe nuclei, and vestibular nuclei (53-gm group).	
			-Electrophysiological: increased amplitude of motor-evoked potentials (53-gm group).	
VLF-ES				
0.32	R (P)	30 days	-Histological: alignment of astrocytes with EF in uninjured white matter. Reduced number of astrocytes and inhibited extension of astrocytic processes within injury site.	72
0.50	R (CT)	84 days	-Histological: increased number of Gal C-positive oligodendrocyte precursor cells (OPCs) and expression of oligodendroglial transcription factors.	76
0.60	R (CT)	84 days	-Behavioral: increased inclined-plane score, modified Tarlov motor grading scale.	75
			-Histological: increased number of axons.	
			-Electrophysiological: reduced motor-evoked potential latency.	
0.40	R (CT)	56 days	-Behavioral: improved Basso–Beattie–Bresnahan (BBB) scores.	78
			-Histological: improved differentiation of OPCs.	
			-Electrophysiological: improved transcranial magnetic motor-evoked potentials.	
0.40	R (CT)	84 days	-Behavioral: improved BBB and inclined-plane scores.	104
			-Histological: enhanced relative area of myelin in spinal cord slices.	
			-Electrophysiological: reduced latency of motor-evoked potentials.	
0.40	R (CT)	35 days	-Behavioral: improved BBB scores.	81
			-Histological: enhanced differentiation of neural stem cells and oligodendrocytes. Faster regeneration of myelinated axons.	
0.14–0.21	D (N)	105 days	-Behavioral (at 6-month follow-up):	69
			Superficial and deep pain: more recovery.	
			Proprioception and locomotion: more recovery.	
			Combined scored: more improvement.	
			-Electrophysiological: more-evoked potential recovery.	
0.50–0.60	D (N)	166 days^ii^	-Behavioral (at 6-month follow-up):	71
			Superficial pain: more recovery.	
			Deep pain: no significant difference between groups.	
			Proprioception and ambulation: no significant difference between groups.	
			Combined scored: more improvement.	
			-Electrophysiological: no significant difference between groups.	
0.50–0.60	H (N)	105 days	Improved recovery of light touch, pinprick sensation, and motor function as compared to baseline.	73, 74

**TABLE IV.** Electrode separation for the various studies. ^i^ Electrode position was adapted to the size of naturally occurred injury during surgery.

Electrode separation	Laminectomy for electrode placement	Reference
Two vertebral segments	No	78, 81–84, 104
	Yes	53, 70, 72
15 mm from injury	Yes	79, 80
20 mm from injury	Yes	29
Individual^i^	No	71, 73, 74
60 ± 21 mm	No	69

In the case of a uniformly distributed field, the electrical field strength *E* at a certain time point equals

$$E=\frac{U_{\text{tissue}}}{l}=\frac{R_{\text{tissue}}I}{l},$$ (4)

where l is the length of the stimulated area, I the current, and $R_{\text{tissue}}$ the resistance between the electrodes. $R_{\text{tissue}}$ can either be measured directly in the tissue or calculated by

$$R_{\text{tissue}}=\frac{\rho l}{A},$$ (5)

where ρ is the ionic resistivity of the tissue, and A its cross-sectional area. Combining both equations leads to

$$E=\frac{\rho I}{A}.$$ (6)

Thus, the field can be estimated from the applied current, the dimensions of the spinal cord, and the resistivity of the stimulated tissue. When measuring the voltage drop or resistance within the spinal cord during an experiment, various factors need to be considered. The input resistance of the measurement system has to be large in order to minimize the current going into it and, thus, bypassing the tissue. The measurement setup has to be calibrated with the measurement electrodes connected to account for their resistance. In addition, the material of the probing electrodes should be non-polarizable (e.g., Ag/AgCl) to deliver a stable readout. Knowing the distance between the measurement electrodes is crucial to relate the measured resistance or voltage to field strength [Eq. (4)].^121^ However, application of the presented equations is not always straightforward. A single resistance, such as $R_{\text{tissue}},$ naturally is a poor model of the spinal cord, because it does not account for different tissue layers, indicated in Fig. 1(a) as parallel circuits with two resistors accounting for transversal (x,y direction) and longitudinal (z direction) current paths. If each tissue layer is represented by only a single longitudinal resistor, this would yield a similar field strength in each layer because various other current paths are disregarded, i.e., damping of transversal current or current passing further dorsal from the cord. Assuming that electrodes are embedded in one tissue layer, e.g., epidural tissue, the applied current does not fully penetrate the white and gray matter, but also leaks into other sections of the cord, such as cerebrospinal fluid or muscle tissue. The magnitude of the leaking current can be minimized by introducing a directionality of the stimulation current via insulation, e.g., toward the dorsal side or by bringing electrodes closer to the cord. Nevertheless, it can be assumed that only a fraction of the stimulation current actually travels through white and gray matter and generates an EF in these tissue layers, which needs to be taken into consideration. In short, for a better estimation of which field strength is generated in a certain tissue layer, ρ and I need a more realistic representation, which is possible to provide by a FEM-model.

**FIG. 2.**
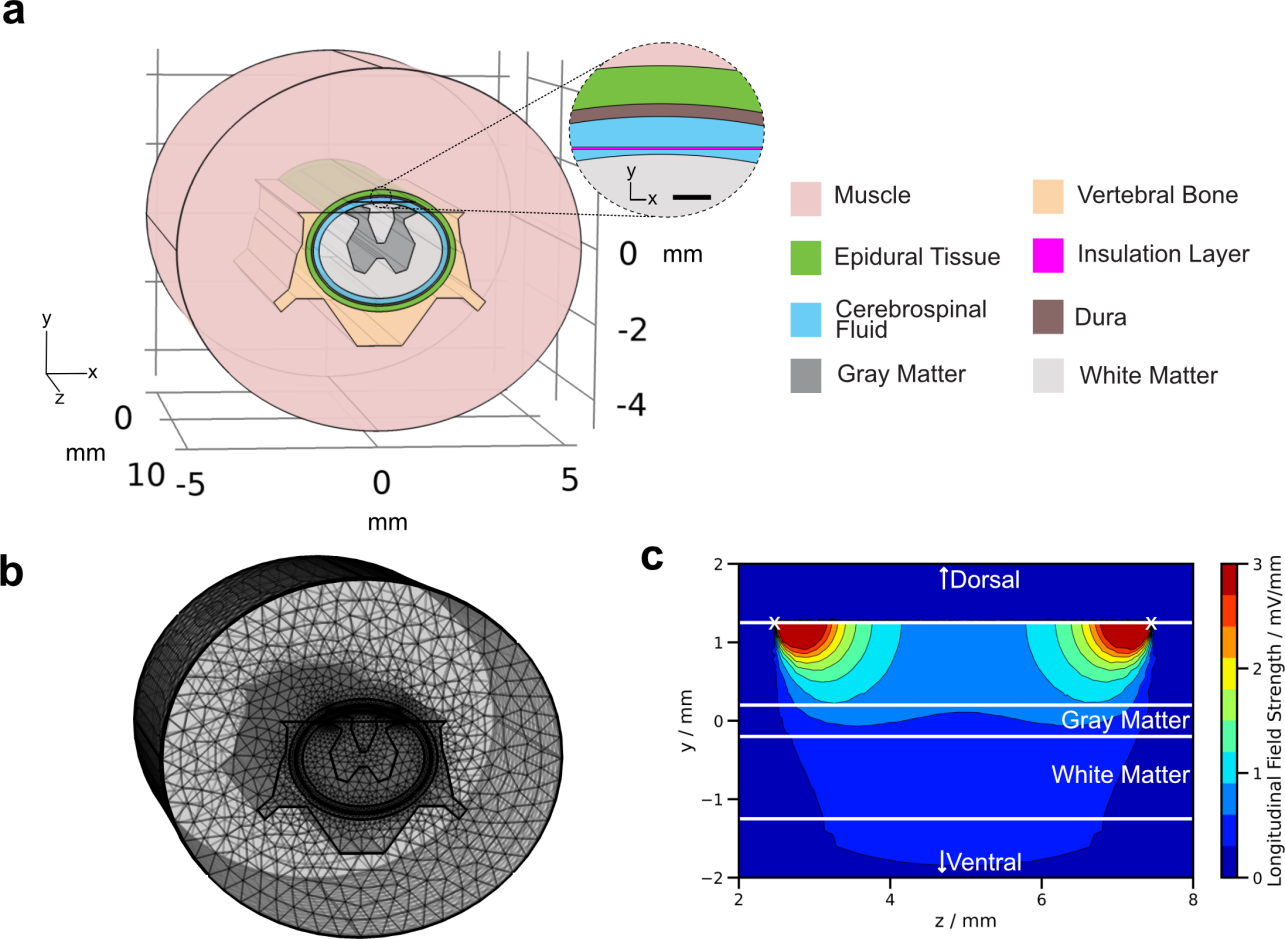
(a) Simple model of a rat's spinal cord with an implant positioned under the dura (insulation layer), in space filled with cerebrospinal fluid (CSF). The scalebar in the inset represents 150 *μ*m. (b) In FEM, the volume is divided into smaller elements on which the equations describing electric field propagation are solved. (c) The computed field strength on the yz-plane (x = 0) in the middle of the spinal cord reveals that the longitudinal field strength generated by two electrodes (white cross) depends on the tissue type and distance from the electrodes.

Cerebrospinal fluid (CSF) has high conductivity and, therefore, shunts the ES, meaning the stimulus will leak in non-targeted regions. A promising concept to circumvent the shunting effect of CSF is to place electrodes subdurally in direct contact with the spinal cord itself.^122^ In order to review this approach, we have modeled a simplified rat's spinal cord [see Fig. 2(a)]. We followed the methods from previous simulations of the human spine^51,52,122^ but placed the stimulation electrodes in the CSF region of the rat's spinal cord according to the work of Harland *et al.*^46^ In general, FEM simulation is based on the idea of dividing the volume into a large number of mesh elements with known geometry to reduce complexity [Fig. 2(b)], thus allowing to account for different tissue layers. As shown in Fig. 2(c), the longitudinal field strength varies along the depth and length of the cord. By placing electrodes subdurally, the EF is concentrated in the spinal cord region between the electrodes. In addition, the EF penetration depth extends to reach the ventral parts of the white matter. Field concentration and penetration depth are smaller for more dorsal electrode positions.^51,122^ The cross section in Fig. 2(c) points out that if values are directly measured in the tissue, it is important to place the recording electrodes in the target tissue because the field strength is not constant in each tissue layer.^51,52^

**FIG. 3.**
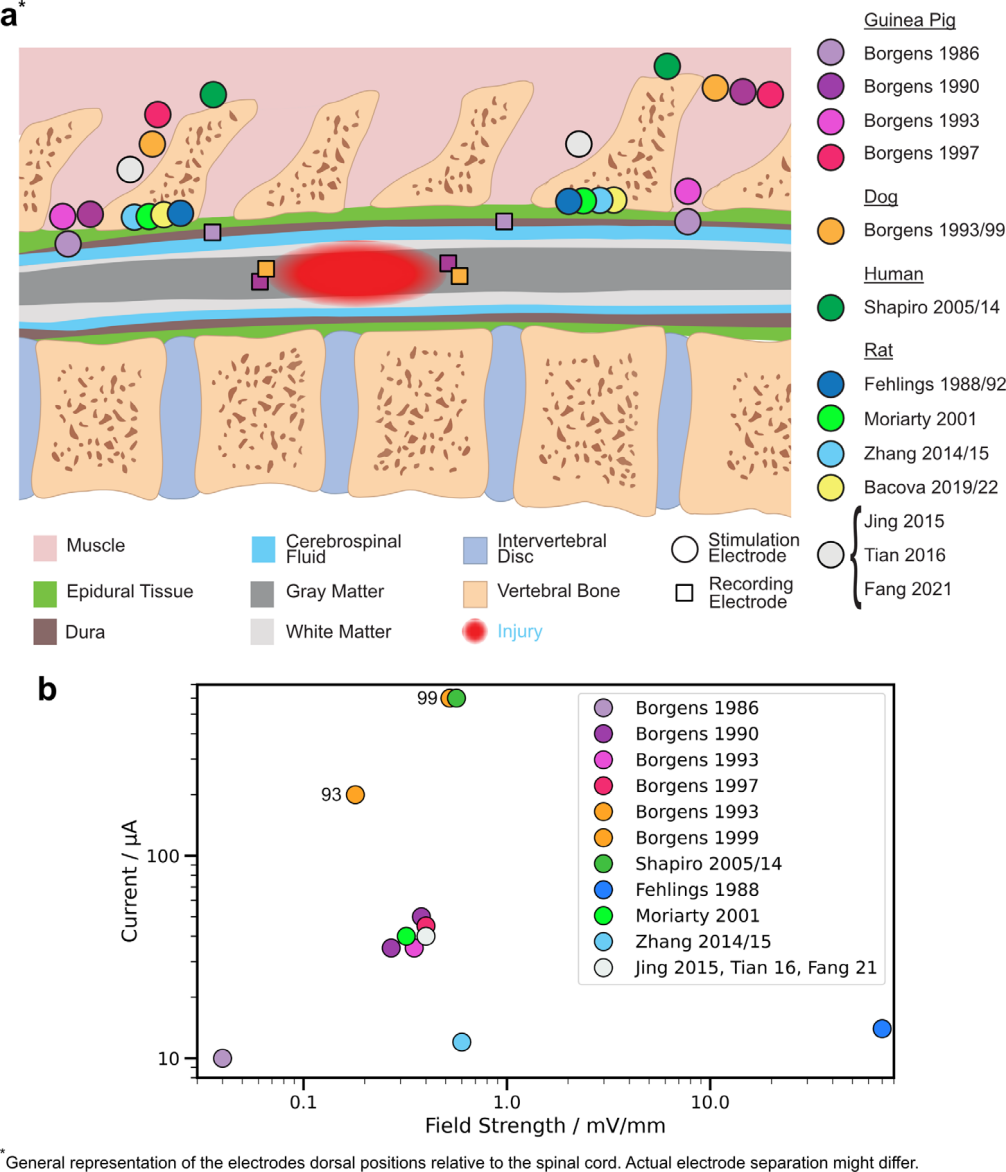
(a) The electrode position in mammals relative to the injury is shown as deduced from publications in which the placement was described. The model of the spinal cord is dimensionless because various animal models (rats, guinea pigs, dogs, and humans) were considered. In some studies, the resulting field strength was measured by recording electrodes (rectangles). In the work of Shapiro *et al.*^73,74^ and Borgens *et al.*,^71^ three sets of stimulating electrodes were utilized; we only show the approximate placement of one electrode pair. More details on electrode separation are given in Table IV. (b) Estimated field strength vs current as stated in the reviewed literature. Circles for Shapiro *et al.*^73,74^ and Borgens *et al.*^71^ were shifted for better visuality; in the works, the same values are reported.

In the following paragraphs, field strengths reported in experimental studies will be outlined. We considered each animal model individually, followed by a critical review of the methods used to estimate field strength in each case. The positioning of the electrodes, the estimated field strength, and the stimulation current if stated are shown in Fig. 3(a).

### A. Lamprey

In studies in lamprey,^63,65^ the generated field strength for 10 *μ*A was estimated to be 10 mV mm^−1^ based on Eq. (6). The stimulating electrodes were not in contact with the spinal cord; therefore, it can be assumed that a current smaller than $I_{S}$ penetrated the spinal cord, resulting in an overestimation of the generated field strength.

### B. Guinea pigs

Borgens *et al.*^29^ measured the resistance of the current path in guinea pig spinal cords in order to calculate the generated electrical field [Eq. (4)]. In addition to the two stimulation electrodes, two recording electrodes were positioned in the CSF above the spinal cord, separated by 20 mm (Table IV.), measuring the resistivity to 73 Ω. Based on this, the field strength at $I_{S}$ at 10 *μ*A was estimated to be 0.04 mV mm^−1^. While this might be true for the generated field in the CSF, a statement about the resulting field strength in the spinal cord cannot be made because only the resistance of the CSF and not the cord itself was measured. Nevertheless, the estimated field strength was furthermore the basis for a follow-up study utilizing 50 *μ*A to generate 0.2 mV mm^−1^. In the follow-up study, the dura was not incised as before.^64^ Thus, results in both studies should be interpreted having this discrepancy between assumed field strength and actual field strength in mind. The influence of stimulation electrodes distally positioned on field strength was investigated by temporarily inserting proximal recording electrodes (injected at 10 mm separation) during the surgery. The voltage difference between the two recording electrodes was measured for various stimulation electrode positions finding that 50 *μ*A generated a field of 0.38 mV mm^−1^ [Eq. (4)] if anode and cathode are placed within a partial laminectomy.^53^ While the current increased by five times, the field strength increased by a factor of 10 compared to the previous measurement in the same animal model but with a slightly different stimulation electrode position [see Fig. 3(a)].^29^ Moreover, it was found that the field strength decreased by 10% (∼0.35 mV mm^−1^) if the anode was positioned outside the vertebral column instead of within a partial laminectomy. In addition to 50, 35 *μ*A was utilized in the same work with an estimated field strength of 0.27 mV mm^−1^. The estimated field strength for the cathode placed within a laminectomy and the anode at the muscle (7.6 *μ*V/mm/*μ*A), which was based on the voltage measurements, was further used in follow-up studies.^70,84^ The point with suturing the anode to paravertebral muscle was motivated by that it reduced the invasiveness of the surgical intervention.

### C. Rats

Hurlbert and Tator.^123^ did not perform SCI treatment but studied the field distribution generated in the rat's spine by extradural disk and cuff electrodes. The field was probed at different positions and depths finding that the generated field strength in the center of the cord between two disk electrodes is approximately constant at 0.26 mV mm^−1^ for 14 *μ*A. Fehlings *et al.*^83^ estimated the generated field strength for 14 *μ*A to be 70 mV mm^−1^ with stimulation electrodes placed extradurally. This is more than 100 times higher than what was estimated in guinea pigs with similar current strength.^29^ Even considering different positioning of electrodes, size of the animal model or distance between the electrodes, the reported field strength must be interpreted with caution. The assessment is based on the measurement of the voltage drop over the entire circuit. Since the voltage does not solely drop over the tissue but also over the two electrode interfaces, the measured value over-estimates the field in the tissue. Moriarty *et al.*^72^ estimated the generated field strength to be 0.32 mV mm^−1^ for 40 *μ*A delivered by electrodes sutured to paravertebral musculature within a laminectomy, referring to the work of Borgens *et al.*^53^ in guinea pigs. A field strength of 0.50and 0.60 mV mm^−1^ was reported for 12 *μ*A with similar electrode placement.^75,76^ The studies unfortunately did not provide a description of how the field strength was determined. A 40 *μ*A stimulation by electrodes sutured to intervertebral muscles was reported to generate 0.40 mV mm^−1^, but unfortunately no further explanation was given.^78,81,104^ In Zhang *et al.*,^124^ 400 *μ*A delivered to extradural electrodes generated a field strength of 270 mV mm^−1^. This estimation is made using Eq. (4), but unfortunately not including the fact that only a fraction of the stimulation current is penetrating the cord itself. In the work of Bacova *et al.*,^79,80^ electrodes fixed to paravertebral muscles 15 mm caudally and cranially from the injury delivered 50 *μ*A. The resulting field strength was not estimated in this case.

### D. Dogs and humans

Dogs are a very common animal model for evaluating spinal cord therapies for human because of the similarity in size of the spinal cord structure and the prevalence of naturally occurring SCI, meaning dogs admitted to veterinary clinics for SCI injury are included in research trials. We will therefore discuss humans and dogs in parallel. Stimulators were first tested in paraplegic dogs before starting with trials in humans. In early work, the electric field generated by two stimulation electrodes (200 *μ*A) positioned at paravertebral muscles on the margins of a hemilaminectomy above the injury delivered a field strength of 0.30, 0.22, and 0.18 mV mm^−1^ for an electrode spacing of 25, 50, and 90 mm, respectively. This was estimated by measuring the voltage drop with two recording electrodes placed within the injury site.^69^ According to Eq. (6), the field strength is independent on the spacing of the electrodes. The decrease measured in practice is likely related to a slightly different positioning at each distance, changing the current within the cord, or by deviations in the measured value related to variable impedance of the recording electrode surfaces. In the same work, it was noted that washing the wound with saline reduced the measured differential voltage and thus field strength. This likely reflects a reduced voltage drop over the electrodes in response to a wetter interface having lower impedance, rather than a reduction of the impedance of the spinal structures, which will not be impacted by saline treatment. This highlights the problems with estimation of the electrical field based on the voltage applied, rather than the current driven through the tissue. Electrode spacing for each individual dog was different, which lead to the estimation of an average field strength between 0.14 and 0.21 mV mm^−1^ for an average electrode spacing of 60 ± 21 mm. In a follow-up study, three electrode pairs were implanted in paraplegic dogs and used for stimulating with a total of 600 *μ*A. Based on the measurement in the prior work, the field was estimated to be three times as high, thus reaching between 0.50 and 0.60 mV mm^−1^.^71^ This estimation could be true under the condition that all electrodes were positioned equidistant in the transverse orientation of the cord. In the first human trial, similar three electrode stimulators were used. The field strength was estimated to be similar to the dog studies but disregarding the difference in spinal cord size and electrode positioning.^74^ Nine years later, the study was continued with four other patients.^73^

The reported field strength in most of the reviewed studies is between 0.20 and 0.60 mV mm^−1^. Biological effects range from improved locomotion to enhanced cell differentiation (see Table III). The effect of axon outgrowth with similar stimuli is usually investigated in cell cultures with a field strength above 5 mV mm^−1^.^26^ Future *in vivo* studies should explore the effect of higher field strengths. Higher field strengths require higher currents, which can cause damage to the tissue. Two mechanisms of damage can generally be categorized. First, damage by the release of cytotoxic stimulation by-products. Hurlbert *et al.*^100^ showed that current densities above 0.75 *μ*A/mm^2^ during 14 weeks of DC-ES delivered by PtIr electrodes caused damage to the spinal cord of rats. The current threshold depends on the electrode placement, the electrochemical reactions, and thus on electrode material and the stimulus used. Electrodes are normally positioned epidurally; however, subdural placement of electrodes reduces the current required by an order of magnitude, which may justify the requirement of a more invasive surgery for DC-ES and VLF-ES treatments.^122^ Materials that mainly inject charge by capacitive or pseudocapacitive mechanisms release less stimulation by-products into the tissue. Monophasic stimulation (e.g., DC-ES) inevitably causes faradaic reactions whose role in charge injection can be reduced by biphasic stimulation (e.g., VLF-ES).^125^ The second proposed mechanism of damage is caused by the overstimulation of the tissue. For short pulses (*μ*s), ES may cause many neurons to fire or neurons to fire over a long period of time, which leads to tissue damage.^126^ The effect of DC-ES and VLF-ES on biological processes is not well understood. More research is required to make a statement about damage by tissue overstimulation through DC-ES and VLF-ES. A common reported stimulation threshold in the literature is 30 *μ*C/cm^2^ based on the first deep brain stimulator approved in the US.^127^ However, stimulation at lower charge densities was shown to induce damage, whereas stimulation at higher charge densities was reported to be safe.^128^ The tolerated stimulation dose is a complex interplay of charge density, charge per pulse, stimulation frequency, electrode material, surface area, morphology, and target tissue. Hence, experiments are needed to assess the effect of a specific stimulation protocol on tissue. Future studies are necessary for a systematic understanding of tissue damage by ES.

In summary, using FEM, it is possible to obtain a more realistic view on current spread throughout the spinal cord and thereby field strength. Through simulations, it was found that the generated field strength in human trials^73,74^ was at most half of the estimated field strength.^51^ The generated field strength may be enhanced by increasing the stimulation current or by bringing electrodes closer to the cord.^129^ The former is challenging for the electrode material due to electrochemical reactions as discussed in Secs. II and III. The latter demands a design that limits the influence of the implant on the cord itself (i.e., compression). As shown in Fig. 2(c), the field strength decreases with distance from the electrodes; thus, bringing electrodes closer to the cord enhances the generated EF in the spinal cord. The subdural position in addition reduces the shunting effect of the CSF, which limits the leakage of current in structures that are not targeted.^122^ Three *in vivo* measurements stand out because recording electrodes were directly inserted into the cord of rats,^123^ guinea pigs,^53^ and dogs.^69^ Therefore, it can be assumed that the measured values describe the generated EF more accurately compared to other studies that used measurements in CSF^29^ or estimations disregarding the fact that only a fraction of the generated current flows through the cord itself.^63,124^ However, a detailed description of the measurement setup would be needed to further assess the measured values. The input impedance of the voltmeter used as well as the DC impedance of the recording electrodes would also influence the accuracy of measurements.^121^ In summary, when evaluating reported field strengths, it is important to also analyze the validity of measurement methods used and which assumptions were made in estimating the field strength in the respective animal model. This first has to be assessed in order to accurately compare studies and interpret results. It is assumed that the axons react primarily to the generated field strength, wherefore accurate information on the actual field strength reached at the axons is pivotal for optimizing the treatment. Medical imaging techniques in combination with FEM simulations can help in improving the accuracy of such field estimations.^18,20,49,50^ The therapeutic effect of the generated EF can be evaluated with different methods, which are explored in Sec. VII.

## VII. ASSESSING THE EFFECTS OF ELECTRICAL STIMULATION TREATMENT ON SCI

This review has focused on studies where the mechanism of recovery is through axonal (and other) regeneration guided or accelerated by an EF. Treatment effects can be studied in animal models through histology, while functional assessments can be conducted in animals as well as humans. Nerve conduction studies can also be used to assess improvements in nerve conduction related to electrical stimulation treatment of SCI. In the future, this method may be applied to clinical patients if stimulation electrodes are combined with recording electrodes in an implantable device.^46,130^ Here, we review and comment on histological, functional, and electrophysiological methods that have been used to assess the effectiveness of electrical stimulation treatments for SCI. We also explore the limitations of studies in a preclinical context. Here, we especially focus on the influence of immediate application of ES after SCI by referring to studies aiming to compensate the initial injury potential through stimulation rather than applying DC-ES over the long term. In combination with an appropriate stimulator design, this supports the experimental planning of future works. The biological effects as effect of generated field strength and treatment duration are shown in Table III.

### A. Histological assessment

Electric fields are applied to damaged tissue in order to promote and direct axon growth; therefore, assessment of axon regeneration is of particular interest. In seminal work using lamprey and guinea pigs, Lucifer yellow and horseradish peroxide (HRP) or conjugated-dextran dyes were applied to the spinal cord at the caudal end of the lesion. Electrical stimulation treatment resulted in more dyed axons growing close to, around, or through the injury site.^29,53,63,65,84^ Furthermore, dye-labeled axons were shown to have enlarged tips, suggesting the presence of growth cones. Subsequent work in rats also applied HRP to the cord caudal to the injury and showed increased counts of labeled neurons in several brain regions (red nucleus, raphe nucleus, vestibular nucleus) after electrical stimulation treatment to the spinal injury.^82,83^ These axon tracing methods allow the visualization of regenerated axons but suffer from certain drawbacks such as the potential for dye leakage, cross reactivity, nonspecific labeling, and a limited time window for detection. Another approach for measuring regeneration is the use of immunoassays to label proteins related to axon growth or differentiation of new neurons or oligodendrocytes. Several studies have shown upregulation of regeneration and differentiation-related molecules around the spinal lesion after application of electrical stimulation using these techniques.^67,68,78,79,81^ Another method to label growth-related proteins, an enzyme linked immunosorbent assay (ELISA), showed similar increases in protein levels after electrical stimulation treatment.^78^ However, ELISA has the disadvantages of requiring a large block of spinal cord to be sacrificed and chemically broken down and any differences cannot be related back to specific regions within the block.

A modern approach to visualize axon regeneration without the drawback of molecular tracers is the use of self-complementary adeno-associated viruses.^131^ These viruses can be injected into the brain to reliably label long-projection axons of interest in the spinal cord. In addition, it is possible to inject a combination of the virus and a tracer molecule to allow separate labeling of sensory and motor axons. It would be of immense interest to re-assess the contribution of electrical stimulation on axonal regeneration after spinal cord injury using these techniques.

Spinal cord injury results in demyelination of axons in the region around the injury; therefore, another focus has been to assess remyelination after electrical stimulation treatment. The amount of myelin and number of myelinated axons in tissue sections can be assessed with Luxol fast blue staining, which has been shown to be increased after ES application at the epicenter and various distances from the injury.^75,79,104,124^ This is likely due to an increased differentiation from stem cells of oligodendrocytes, the cells responsible for myelinating axons, as well as an increased rate of survival and maturation of these cells.^76,78,81^

An inflammatory response of astrocytes is also a hallmark of spinal cord injury resulting in apoptotic cell death and the eventual formation of a glial scar around the injury. Astrocytes labeled with GFAP were significantly reduced in the spinal cord of guinea pigs after 4 weeks of ES treatment, especially in the lesion area.^72^ Densitometric analysis of immuno-stained astrocytes showed a reduction in both the dorsal and lateral columns extending several segments either side of the spinal cord injury in rats treated with electrical stimulation.^79^ In another study, hematoxylin and eosin staining showed that electrical stimulation reduced the number of infiltrating inflammatory cells in the lesion site compared with controls.^81^ Detection of calpain, a protease that triggers cell death post-injury, and a DNA detection method for apoptotic cells both showed reduced levels in ES-treated rats after spinal cord injury.^75^

Treatment with ES has been shown to promote axon regeneration around and through SCI as well as having other beneficial effects such as encouraging remyelination and reducing the neuroinflammatory response of astrocytes. Future studies should continue to investigate the effects of ES treatment on these different pathways as it is likely to be the combination of these beneficial effects that contributes to improvements in functional outcomes.

### B. Functional assessment

The focus in preclinical studies has been various measures of hind leg function after electrical stimulation treatment and SCI. Early studies primarily used the inclined plane test, which identifies the steepest angle at which animals can maintain body balance by the strength of their forelimbs and hindlimbs.^132^ However, most of the studies reviewed here used a thoracic injury model resulting in hindlimb dysfunction only, although some try to minimize the effect of the noninjured forelimbs on this task by positioning the rat lateral with respect to the long axis of the inclined plane.^82^ It is also unclear the exact nature of function tested by this task as it includes elements of grip strength, balance, and sensorimotor function. More recent studies have used the Basso–Beattie–Bresnahan (BBB) scale, a 21-point open field locomotion score for hindlimb function.^133^ The scale is nonlinear, the lower scores concern gross aspects of locomotion, whereas higher scores (≥ 13) concern subtle and discreet movement aspects, which are more prone to inter-rater differences.

Functional recovery after SCI is complicated as different tracts within the cord modulate subtly different types of physiological function. For example, coordinated stepping patterns in normal locomotion are activated by the ventrally located reticulospinal tract, whereas placing response of paws required when walking on a grid or ladder use corticospinal or supraspinal circuits depending on the animal.^134,135^ It is therefore advisable to use a combination of locomotor, sensorimotor, kinematic, and footprint analysis tasks toward a more comprehensive analysis of injury and recovery in SCI.^136^ In contrast, only a few of the preclinical studies investigating electrical stimulation treatment have used more than one functional task.^104,124^

Assessing improvements in sensory function in preclinical studies and not just locomotor function is also critical moving forward. A study in which patient dogs were implanted with stimulators found significant improvements in superficial and deep pain response below the lesion compared with controls, whereas neurologic scores for ambulation and proprioceptive placing were not significant.^71^ Similarly, human SCI patients treated via implanted stimulators had significantly improved light touch and pinprick sensation alongside improved motor scores after electrical stimulation treatment.^73,74^ These results may not be surprising considering that electrodes are always positioned above the dorsal spinal cord, which contains the majority of sensory tracts. Therefore, assessments of sensory function may be more sensitive and capable of detecting improvements sooner after electrical stimulation treatment in preclinical work. Von Fey tasks are an established method to assess the tactile sensation of hindpaws and can also be used to evaluate neuropathic pain. Various tasks for hot and cold sensation are also suitable, such as the Hargreaves test, which assesses pain sensitization or recovery of thermal pain response following neural injury and regeneration. Only a few older preclinical studies investigating electrical stimulation treatment have included a sensory assessment^53,64,70^ in which cutaneous trunci muscle sensitivity below the injury was assessed in guinea pigs.

### C. Electrophysiological assessment

Electrophysiological methods provide an alternative strategy to assess spinal cord function by measuring the integrity of nerve conduction across the injury. Electrodes are placed either side of the injury, usually in the hindlimb muscle and skull or brain surface but sometimes along the spinal cord itself. In the early rat studies, motor evoked potentials (MEPs) were recorded from hindlimb muscle, and sensory-evoked potentials (SEPs) were recorded from somatosensory cortex in terminal surgeries at 8-weeks post-injury.^82,83^ MEPs were recorded in a higher proportion of animals from the electrical stimulation group and at a higher amplitude than control SCI and sham animals, whereas proportion and amplitude of SEPs was similar between groups.

In the studies using patient dogs, electrophysiology was performed under anesthesia at 6 weeks and 6 months post-injury.^69,71^ In the first study, recording needles were inserted transcutaneously into the vertebral space to record stimulation of the hindlimb tibial nerve (spinal-evoked potentials) as well as standard SEPs and MEPs recorded in vertebral space in response to transcranial stimulation. At 6 weeks, one ES-treated dog showed detectable spinal evoked potentials. However, at 6 months, four of the stimulation treatment animals showed recovery of evoked potentials, three of which included recovery of SEPs.^69^ In the follow-up study, only SEPs were tested with 20% of ES-treated dogs, showing recovery at 6 weeks, and 50% at 6 months compared to 15% and 14%, respectively, in controls.^71^ The use of SEPs to test nerve conduction was carried over to the subsequent clinical studies in which patients were re-tested at 12 months after showing minimal or no conduction at the time of stimulator installation. In the first study, three out of five patients with cervical SCI had improvements in arm SEP, with one showing minimal recovery of tibial SEP. Only one out of five patients showed minimal improvement in right leg SEPs at 12 months.^74^ In the second study, six out of eight patients with cervical SCI had improvements in arm SEPs and one recovered tibial SEP, whereas one out of six patients with thoracic SCI recovered minimal right leg SEP.^73^ These improvements were encouraging, but these studies did not have a control group for comparison.

Several more recent studies in rats have used weekly or bi-weekly MEP recordings to assess ES-treatment related recovery.^75,78,104^ In one study, the latency and amplitude of MEPs recorded in the hindlimb via transcranial stimulation was significantly improved in ES-treated rats from week 2 onward compared with SCI controls.^78^ In two other studies, stimulation was compared from electrodes positioned above and below the level of SCI and recorded in the hindlimb to assess conduction through across the injury.^75,104^ In both studies, rat treated with ES had significantly decreased latency of MEPs at 4 weeks onward compared with SCI controls.

Electrophysiology assessment of nerve conduction across the injury has proven to be an effective method to detect recovery after ES-treatment of SCI. These methods complement functional testing, but consideration must be given for the need to anesthetize animals and use invasive needle electrodes to stimulate and record. Moreover, higher stimulation intensity can provide clearer results but must be applied carefully to avoid side effects such as twitching of limbs, head, or ears.^137^

### D. Compensating injury potential

Immediately following SCI, rapid axonal dieback from the injury site occurs over a period of hours. The molecular mechanism responsible is a marked increase in the movement of positive ions toward the injury, which builds up in the damaged or severed axon tips. This was first demonstrated in transected lamprey spinal cord, in which an increased “injury potential” of 300–700 *μ*A cm^−2^ was recorded immediately after the injury from the dorsal surface of the injury site using an ultrasensitive vibrating probe.^138^ This increased flux of positive Na^+^ and Ca^2+^ ions dropped to 100 *μ*A cm^−2^ within 6 h post-injury and reduced further to around 40 *μ*A cm^−2^ the next day where it stabilized for the 6 days of recording. Borgens *et al.*^63^ were able to attenuate the axon dieback by immediately imposing an electric field across the injury with the anode positioned close and cathode distal from the injury. The ES compensated for the injury potential by attracting positive ions away from the injury site, reducing axon degeneration caused by the increased intercellular concentrations of calcium. This same pattern of injury potential in which a marked increase in positive ion current gradually subsides within hours has been shown in mammals including cats, guinea pigs, and using spinal injury models other than transection.^77,139,140^

Several studies have delivered a short 15–45 min ES treatment directly after contusion SCI in rats with a particular focus of normalizing the injury potential via micro-adjusting the injected current to return the potential to a pre-injury level.^77,124,141^ This significantly reduced the effect of the secondary injury cascade by profoundly attenuating lesion size and improving functional recovery. Most other *in vivo* studies have installed stimulator units, which were switched on shortly after the injury.^29,53,64,66–69,71,72,75,76,78–84,104^ The ES treatment delivered via these units was not specifically directed to normalize the injury potential but would reduce the negative effects to some degree. From a clinical perspective, it is not possible to compensate for the injury potential as this would require an electrical field treatment to be administered within minutes or hours of injury onset. Instead, electroceutical treatment might realistically be initiated days, weeks, or years afterward in human patients, aimed at promoting regeneration across the later stage injury. Therefore, interpretation of the results of these studies in a preclinical context is problematic as it is impossible to separate any positive effects of the treatments that may be related to compensation of the injury potential in the first 24 h vs later regeneration of the injury. Future *in vivo* preclinical studies should address this issue by only initiating ES treatment at least 24 h after the injury as this is sufficient time for injury potential to stabilize and also represents a more realistic earliest time point that an electrode treatment might be initiated in a clinical setting.

## VIII. CLINICAL TRANSLATION

Implantable systems are commercially available to deliver ES to the spinal cord. These implants are usually positioned epidurally and incorporate several electrodes. The number of electrodes available has increased over the years allowing for complex stimulation patterns and enhanced precision of the applied stimulation. The main clinical use of spinal cord stimulation (SCS) currently is the reduction of intensity, frequency, and duration of pain (e.g., treatment of failed back surgery syndrome and complex regional pain syndrome).^142^ The capability of similar systems is additionally leveraged in recent clinical trials to induce muscular contraction to carry out functional tasks (i.e., walking, canoeing).^12,14,18^ The electrical stimuli to reduce pain or for activity-dependent neuromodulation usually consists of pulses with frequencies between 20 and 100 Hz.^143^ We guide the reader to the works in Refs. 32, 142, and 143 for a detailed discussion about the use of electrical stimulation for spinal cord injury.

The clinical translation of DC-ES and VLF-ES faces challenges that are unique. Other challenges also exist in currently available neuromodulation systems. The applied electrical stimuli in today's usage of SCS is different to DC-ES and VLF-ES. The application of DC induces large electrochemical stress at the electrode material, which makes DC-ES and VLF-ES especially challenging. For the translation to clinical trials, the effect of the generated by-products in the tissue needs to be evaluated. Commercially available systems for SCS may not be suitable to deliver DC because they are not developed to withstand redox reactions over a long period of time. Implants need to be developed, which incorporate larger electrodes that consist of materials suitable to generate DC (see Sec. III). This means the biocompatibility of the stimuli itself and the electrode and implant material need to be thoroughly studied. Subdural placement of the electrodes directly on the spinal cord would allow a stronger and deeper penetrating electrical field while requiring much less current. These benefits come at the expense of additional surgical risks, which would be justified if the treatment could reliably restore lost function. The development of a suitable stimulator for DC-ES and VLF-ES is costly which is presented as one of the reasons why the phase 1 human trial has not been followed up with a phase 2 trial.^73^ That is, the clinical version needs to be safely sealed, and must include fail safes and cutout circuits. Other aspects relevant for clinical translation are, in general, valid for all types of SCS. First, physicians and patients need to be aware of existing treatment possibilities, and the surgical expertise to safely implant such devices is also required. The therapy then must be tailored to the patient. Since it would be critical to minimize the time between injury and implantation, the course of these events should be streamlined. SCS is most successful when the social environment of the patient and mental health experts are included in the therapy plan.^142^ Post-monitoring of the implant is required to ensure continued functioning and identify any post-surgical complications as early as possible. The design of a clinical trial comes with challenges unique to SCI itself, such as the heterogeneity of the condition. The effect of SCI is individual and complex, which makes the assessment of just one treatment method difficult.^144^ Looking to the future, DC-ES and VLF-ES to guide axonal outgrowth may be combined with other therapeutical methods to alter the biological environment of the spinal cord. While the regeneration capacity of the CNS is poor, it can be improved by neural cell transplantation^20–22^ and pharmacological treatments^23,24^ alongside electric field therapies.

## IX. CONCLUSION

The application of DC to regenerate axons after spinal cord injury is a promising therapeutic approach. Further studies are needed to explore its full potential and central to these studies will be designing stimulation paradigms so that reproducible and effective field strengths are reached. By optimizing implant design, electrode design, and stimulation circuitry, it is possible to administer stimulation with high spatial and temporal control. Imaging and computational models can also support planning of stimulation so that signals can be tailored to the specific anatomy.

As reviewed here, the literature to date describes a diverse set of animal models, stimulators, and electrodes, from which it is not always straightforward to reconstruct which field strength was effectively seen by the target tissue based on the experimental descriptions provided. Taking pharmaceutical interventions as example, it is well accepted that dosage is of exceptional importance for the success of a therapy. We argue that it would be of the utmost importance to apply the same principle for application of DC and continue from here with a common way to describe and monitor experiments. Improved comparability between studies will ensure that every experiment can contribute to knowledge building and accelerate the translation of DC-ES and VLF-ES to clinical application.

An individual, patient-specific approach to ES can be achieved by standardizing electrode placement and reducing between-study/subject variability using FEM modeling based on medical imaging. We have explored the impedance variability of tissue and electrodes, which means stimulator devices need to be engineered to be adaptive and tailored to the target tissue and to be resilient to such variation. It is also important to ensure fail-safe circuitry and to allow for an assessment of the electrode status during the experiment. Thanks to new developments in electrode materials, e.g., the use of pseudo-capacitive materials such as CPs, we foresee a future where stimulation electrodes can be placed closer to the lesion site increasing precision of the treatment, without jeopardizing tissue health.^43,57^

The success of a treatment needs to be carefully evaluated through histology, functional, and electrophysiological assessment. For the latter, an implantable system comprising recording and stimulation electrodes is beneficial. Histology may be used as direct proof of regenerating axons across the injury site, and to identify the specific tracts and circuits that regenerate. Modern approaches such as the use of self-complementary adeno-associated viruses^131^ can be applied to distinguish between motor and sensory fibers. For a patient, the ultimate measure of recovery will be functional improvements. Finally, while most preclinical studies are conducted in the acute post-injury phase, most clinical uses will likely be at later timepoints, therefore preclinical outcomes must be extrapolated with caution. The potential of electrical stimulation to achieve tissue regeneration following SCI is exciting, with recent technology developments opening up new possibilities around electrode placement and performance. We conclude that, the systematic design and interpretation of preclinical research will be essential to translate treatments to the clinic.

## X. METHODS

### A. Stimulation with PtIr wire

Stimulation with a PtIr wire (Pt 90%/Ir 10%, 0.18 mm diameter) is presented as a show case for electrode degradation during DC-ES. The insulation on 5 cm of the wires' tip was removed, which acts as anode. On the other side of the wire, 1 cm of insulation was removed to allow connection to a potentiostat (PGSTAT 204, Metrohm Autolab B.V., Filderstadt, Germany) with an alligator clip. The tip was put into 0.01 M phosphate-buffered saline (PBS, Sigma Aldrich, Germany). The voltage during galvanostatic stimulation (24 h, 17.5 *μ*A) in three electrode setup with stainless steel (∼28 cm^2^) as counter and Ag/AgCl electrode (Ag/AgCl, BASI, USA) as reference was measured. Cyclic voltammetry (100 mV s^−1^) before and after stimulation in new electrolyte solution was recorded in the same electrochemical cell setup.

### B. Finite element simulation of electrical field distribution in spinal cord

FEM analysis is presented to show the complexity of EF distribution in the spinal cord. COMSOL Multiphysics® software (version 5.3) was used to simulate the EF distribution in the spinal cord during DC-ES. A simplified model of a rat's spinal cord was modeled in COMSOL as seen in Fig. 2(a). Dimensions are adapted from the literature.^145–147^ White matter was modeled as an elliptical cylinder with radius 1.625 and 1.25 mm. The width of CSF, dura mater, and epidural tissue were 0.15, 0.05, and 0.15 mm, respectively. The shape of the vertebral bone and gray matter were adapted from atlas segments.^147^ The surrounding muscle body has radii of 5.325 and 4.75 mm. Conductivity and permittivity of the various tissues were used from the work of Hernández-Labrado *et al.* (Table I).^51^ Four electrodes in total were positioned in the CSF region (two rostral and two caudal), with electrode separation of 5 mm in z, and ± 0.25 mm in x from the center of the spine. The electrodes were 300 nm thick Pt (material properties from COMSOL) sheets (60 × 60 *μ*m^2^). 5 *μ*A DC was applied to the two rostral electrodes while defining the other two electrodes as electrical ground. On top of the electrodes an 8 *μ*m thick, 1.8 mm wide insulating polyimide sheet (conductivity = 1.5× 10^−17^ S/m; relative permittivity = 3.4),^148^ representing the implant encapsulation was modeled. For validation, the resulting field distribution was compared to previous works.^51,123^ Due to differences in electrode positions, applied current, interelectrode distance, and shape and dimension of the electrodes, the relative longitudinal field strengths at dorsal (1 mm from spinal surface), central, and ventral (1 mm from spinal surface) level (all at x = 0) were juxtaposed. As reported in previous works, the field strength at the dorsal level drops sharply toward the region between the stimulation electrodes reaching a minimum of approximately 25% of the field strength at the position of the electrodes.^123^ In addition, it was reported that in rodents the central and ventral field strengths at the position between the electrodes are approximately 80% and 60% of the dorsal field strength at the same position, respectively.^123^ The dorsal and central field strengths reach a local minimum between the stimulation electrodes. In contrast, the ventral field strength shows a maximum.^51,123^ In our model, evaluation of the simulated field strengths resulted in comparable curve shapes: the dorsal field strength drops to 26% of the maximum; central and ventral field strengths between the electrodes are 79% and 61% of the dorsal field strength at the same position, respectively. The color map in Fig. 2(c) shows a similar pattern as in the work of Greenbaum.^52^

## Data Availability

The data that support the findings of this study are available from the corresponding author upon reasonable request.
